# Psychometric validation of the short-form Swedish “attitudes to and knowledge of oral health” (S-AKO) questionnaire in Chinese nursing professionals: a cross-sectional study

**DOI:** 10.1186/s12903-026-08076-1

**Published:** 2026-03-13

**Authors:** Xuancheng Chen, Maria Snogren, Linan Cheng

**Affiliations:** 1 School of Nursing, Soochow University, Suzhou, 215006 China; 2 School of Nursing, Hangzhou Normal University, Hangzhou, 311121 China; 3 School of Health Sciences, University of Skövde, Skövde, SE-541 28 Sweden; 4 School of Nursing, Wenzhou Medical University, Wenzhou, 325035 China

**Keywords:** Oral healthcare, Knowledge, Attitude, Practice, Registered nurses

## Abstract

**Background:**

Strengthening nursing professionals’ oral health–related knowledge, attitudes, and clinical practices is essential for improving patient outcomes. While the Swedish Attitudes to and Knowledge of Oral Health (S-AKO) questionnaire is a well-established tool, its validity in China remains unknown. Accordingly, the present study was designed to adapt the S-AKO to the Chinese context and to examine its psychometric properties among nursing professionals.

**Methods:**

A convenience sample of 558 nursing professionals was drawn from three universities and twelve tertiary hospitals across China between September and October 2025. The short-form Swedish S-AKO questionnaire was translated following Brislin’s model, and its Chinese version was administered to collect data. Item analysis, reliability, and validity were assessed with SPSS version 25.0 and AMOS version 24.0.

**Results:**

Following item-level screening, 13 items were retained for inclusion in the Chinese adaptation of the S-AKO. The instrument demonstrated satisfactory reliability, including internal consistency (Cronbach’s α = 0.810), split-half reliability (0.726), and temporal stability (test–retest reliability = 0.923). Content validity was well supported, as indicated by an item-level CVI of 0.857 and a scale-level CVI of 0.907. Exploratory factor analysis (EFA) revealed a three-factor structure: “Attitudes to Oral Hygiene,” “Implementation Possibilities,” and “Knowledge of Importance,” which together accounted for 80.94% of the variance observed in the data. Subsequent, confirmatory factor analysis (CFA) provided further evidence for the adequacy of the proposed model, demonstrating acceptable model fit (CMIN/DF = 2.466, RMSEA = 0.073, CFI = 0.970, IFI = 0.970, NFI = 0.951, TLI = 0.962).

**Conclusion:**

The adapted S-AKO in Chinese demonstrated acceptable validity and reliability among nursing professionals. Its application may inform adjustments to nursing curricula and support the integration of evidence-informed oral care in education and clinical practice.

**Supplementary Information:**

The online version contains supplementary material available at 10.1186/s12903-026-08076-1.

## Introduction

It is estimated that around 3.5 billion individuals globally experience oral health–related conditions [1], with dental caries and periodontal disease constituting highly prevalent chronic conditions that remain largely preventable [2]. Poor oral health often leads to malnutrition, speech and swallowing difficulties [3]. Moreover, an expanding body of research has identified robust links between oral health status and a range of systemic conditions, including cardiovascular disease, aspiration pneumonia, and diabetes [4, 5]. Moreover, compromised oral health increases overall morbidity and mortality, prolongs recovery time [6, 7], and negatively impacts individuals’ psychosocial functioning [8]. In contrast, maintaining good oral health reduces the risk of infection and related complications, serving as a key foundation for achieving healthy ageing [9].

Given these far-reaching consequences, promoting oral health is no longer the sole responsibility of dental professionals but requires collaborative efforts across healthcare disciplines [10, 11]. Globally, increasing attention has been directed toward strengthening interdisciplinary collaboration in oral healthcare [12, 13], particularly through the active involvement of non-dental professionals such as nurses. Such efforts aim to enhance awareness of oral health, promote preventive behaviours, and ultimately improve oral health outcomes. Nursing professionals, including both nursing interns and registered nurses, are central to the evaluation of oral health in hospitalized and care-dependent patients through oral assessment, dental referral, and interdisciplinary collaboration, which have been shown to improve both oral and overall health [14, 15]. However, previous research has revealed that nursing professionals often receive limited training and education in oral health and oral healthcare [16, 17]. This educational gap not only leads to insufficient knowledge and misconceptions but also results in a lack of confidence, lower perceived feasibility, and reduced motivation to implement oral healthcare in clinical settings.

Deficiencies in oral healthcare knowledge emerge early in nursing education and are exacerbated by the growing demand for highquality oral care practices alongside students’ persistent lack of engagement with this domain [18]. This situation has led to an increasing discrepancy between expectations for advanced clinical competence and actual performance, indicating a disconnect between curricular objectives and their translation into clinical practice [1, 2]. Specifically, this misalignment manifests as a disconnect among knowledge, practical skills, and attitudinal readiness. When education fails to integrate knowledge acquisition, attitude formation, and practice translation, observable deficits arise: nurses may cognitively acknowledge the importance of oral care, yet remain constrained by inadequate attitudinal preparation, low selfefficacy, and negative perceptions of feasibility, hindering the translation of knowledge into consistent and competent clinical practice [10, 19]. According to the Theory of Planned Behavior, behavioral change depends not only on knowledge but also on attitudes, subjective norms, and perceived behavioral control [20]. Thus, evaluating nursing professionals including both interns and registered nurses in terms of their oral health knowledge, attitudes, and perceived capacity for implementation can help elucidate the mechanisms underlying the theorypractice gap in oral healthcare and identify targeted interventions to bridge the divide between educational objectives and clinical practice.

From a competency-based perspective, nursing competency in oral healthcare can be operationally defined as a multidimensional construct encompassing cognitive understanding of oral health importance, attitudinal orientation toward oral hygiene, and perceived feasibility of implementing oral care in routine clinical practice [21]. Drawing on established competency frameworks in nursing education and healthcare practice, this construct emphasizes not only cognitive mastery of oral health principles but also the motivational and practical dimensions required for effective clinical application. Previous studies have highlighted that deficiencies in any of these domains may undermine oral healthcare delivery, even when nurses are theoretically aware of its importance [22–24]. Accordingly, valid assessment of oral healthcare competence requires instruments capable of capturing these distinct yet interrelated dimensions, rather than treating oral care as a unidimensional skill or knowledge domain.

In the Chinese context, content related to oral health is commonly embedded within foundational nursing courses, geriatric nursing, or community health nursing, with considerable variation in depth and practical exposure across institutions [25]. As a result, nursing students may acquire fragmented theoretical knowledge without sufficient opportunities to consolidate learning through structured skills training or supervised clinical application, leading to an incomplete internalization of oral healthcare competencies. In routine clinical nursing practice, oral care is generally recognized as a fundamental nursing responsibility [26]; however, its implementation is often influenced by institutional priorities, workload constraints, and the availability of standardized training and assessment tools [27–30]. These contextual constraints can impede the systematic incorporation of oral healthcare within everyday nursing care delivery.

Several instruments have been developed internationally to assess these dimensions, though each has its limitations. In China, Cheng et al. conducted the cross-cultural adaptation and psychometric evaluation of the Attitude and Confidence with Oral Healthcare among Nursing Students (ACORN) scale, which measures nursing students’ perceptions of and confidence in oral healthcare [27]. The Chinese adaptation showed satisfactory psychometric performance. However, it does not include an assessment of oral health knowledge and has not been validated among clinical nurses. Additionally, the ACORN scale, with its 22 items, requires respondents to complete a relatively longer questionnaire, which may increase completion time and cognitive effort. Methodological research on questionnaire design has shown that longer instruments are more likely to be associated with increased respondent burden, lower response rates, and reduced feasibility, particularly in large-scale surveys or busy clinical settings [31]. Consequently, the practical utility of the ACORN scale may be limited when repeated or routine assessments are required.

Building on the original Attitudes to and Knowledge of Oral Health (AKO) questionnaire proposed by Paulsson et al. [32]. Snögren subsequently derived a concise Swedish short-form, known as the Swedish Attitudes to and Knowledge of Oral Health (S-AKO) questionnaire [33]. This 13-item short form is concise, user-friendly, and demonstrates strong psychometric properties among 611 nurses, effectively assessing attitudes toward oral hygiene, perceived possibilities for implementation, and knowledge of the importance of oral health. It offers practical advantages over the longer ACORN scale and addresses its limitations.

There is a pressing need for an assessment tool that is both psychometrically robust and culturally appropriate for the Chinese context. China’s aging population also fuels a growing demand for oral healthcare [34], highlighting the importance of a validated instrument for systematically assessing nursing professionals’ knowledge and attitudes. This tool is intended to support the assessment of nursing professionals’ oral health–related knowledge and attitudes across educational and clinical contexts. To enhance transparency and facilitate interpretation, an item-level overview detailing the content of each domain of the S-AKO is provided in Appendix 1.

## Methods

### Design and participants

The Swedish S-AKO questionnaire was translated into Chinese, and its reliability was examined in a multi-site nursing sample. From September to October 2025, a convenience sample of 318 undergraduate nursing interns enrolled at three universities in Hangzhou and Wenzhou, Zhejiang Province, and 240 registered nurses (RNs) from 12 tertiary hospitals across Jilin, Shandong, Jiangsu, Tianjin, Chongqing, and Zhejiang were recruited.

Nursing interns were eligible if they were enrolled as full-time undergraduate students and provided voluntary informed consent to participate. Exclusion criteria included internship interruption due to illness, personal reasons, or leave, as well as declined or discontinued participation. RNs were eligible if they possessed a valid nursing license, were actively employed in clinical practice, and provided informed consent. Individuals not currently practicing, including those on leave or external training assignments, or those who declined participation, were excluded.

An extra convenience group of 30 eligible participants, comprising both nursing interns and RNs, was recruited independently to evaluate one-week test–retest reliability. This subsample was not included in the main analyses to preserve sample independence.

### Instruments

The demographic questionnaire collected data on gender, age, residence, passion for nursing (defined as participants’ self-reported intrinsic professional interest and emotional commitment to the nursing profession) [35], use of oral care products, oral healthcare training (defined as any formal or informal education related to oral health received during nursing education or clinical practice) [36], and self-rated oral health (assessed using a single global item evaluating participants’ overall oral health status) [37] from both groups. Additionally, it gathered information on educational attainment, years of experience, professional title, and current department from the RNs.

The Swedish short-form Attitudes to and Knowledge of Oral Health questionnaire (S-AKO), proposed by Snögren et al. in 2022 [33] (See Appendix 2), was derived from the original AKO instrument developed by Paulsson et al. [32]. The instrument is designed to assess healthcare professionals’ oral health–related attitudes and knowledge. It consists of 13 items distributed across three domains: Attitudes to oral hygiene (3 items), Implementation possibilities (4 items), and Knowledge of importance (6 items). Responses are recorded on a five-point Likert scale. The first two dimensions are rated from “never” (1) to “always” (5), whereas the third dimension ranges from “not important” (1) to “important” (5). Possible total scores span from 13 to 65, with higher scores indicating more favorable attitudes and higher levels of oral health knowledge. The instrument has previously demonstrated acceptable reliability, with Cronbach’s α coefficients across the three subscales ranging between 0.460 and 0.869 [21].

### Translation procedure

With permission obtained from the original developer, the S-AKO questionnaire was translated and culturally adapted in accordance with Brislin’s model [38]. Two bilingual postgraduate nursing students independently completed the forward translation from English to Chinese, generating two preliminary versions (A1 and A2). These were discussed and merged by the research team into a reconciled version, A3. Two blinded translators then performed back-translation: one was a nursing faculty member specializing in medical English, and the other a postgraduate student in English translation. Their back-translations, B1 and B2, were consolidated into a single English version, B3. The original author reviewed B3 to assess semantic and conceptual equivalence. Based on the author’s feedback, wording was refined, and the translation–back-translation cycle was repeated until no substantive discrepancies persisted, resulting in the preliminary Chinese S-AKO.

Content validity was assessed by a multidisciplinary expert panel (*n* = 14) with expertise in geriatric nursing, nursing education, oral nursing, and nursing management. Each item was evaluated for relevance and representativeness using a four-point Likert scale. Expert feedback informed revisions to the Chinese draft. Subsequently, a pilot study with 30 nursing interns and RNs was conducted to examine clarity, cultural suitability, and conceptual consistency [39]. As no additional modifications were deemed necessary, the finalized Chinese version of the S-AKO questionnaire was confirmed.

### Data collection

Data collection was conducted via Wenjuanxing, a Chinese online survey platform. After obtaining institutional consent, eligible participants received the survey link through WeChat or DingTalk. The questionnaire included a study introduction, informed consent form, and the Chinese S-AKO version. Electronic consent was required to proceed, with an emphasis on voluntary participation and anonymity.

Submitted responses were screened based on the following criteria: completion in under 120 s, inconsistent answers, or repetitive patterns. Sample size was estimated using Kendall’s method [40], recommending 10–15 participants per item. With 13 S-AKO items and a 20% attrition rate, 156–234 participants were initially required. However, in accordance with methodological recommendations for confirmatory factor analysis, a minimum sample size of 400 was predetermined.

### Statistical analysis

Statistical analyses were performed with IBM SPSS Statistics 25.0 and AMOS 24.0. Given the differences in educational background and clinical experience, nursing students and RNs were described separately to provide a clearer characterization of the sample. Descriptive statistics for nursing interns and RNs were reported separately, with continuous variables summarized as means with standard deviations or medians with interquartile ranges, and categorical variables expressed as frequencies and percentages. Group differences were examined using independent-samples t tests, chi-square tests, Mann–Whitney U tests or Kruskal-Wallis Htests as appropriate. A two-sided *p* value < 0.05 was considered statistically significant. Exploratory and confirmatory factor analyses were subsequently performed on the combined sample to evaluate the underlying factorial structure of the instrument across different professional stages.

### Item analysis

Item analysis assessed the discriminative power and alignment of each item with the overall measurement objectives, using critical ratio (CR) statistics and item–total correlation analyses. Participants were ordered according to overall scale scores, and the upper and lower 27% were designated as high- and low-performing groups. Group differences were tested using independent-samples t tests; items achieving CR values of at least 3.0 with statistical significance (*p* < 0.05) were retained [41]. In addition, associations between individual items and the total scale score were evaluated using Pearson correlation coefficients. Items showing correlations of 0.30 or higher (*p* < 0.05) were considered acceptable for inclusion in the final instrument [42].

### Validity analysis

#### Content validity

Content validity was assessed by a panel of 14 subject-matter experts using both item-level (I-CVI) and scale-level (S-CVI) content validity indices. Experts rated the relevance of each item on a four-point Likert scale ranging from 1 (not relevant) to 4 (highly relevant). In accordance with established criteria, content validity was considered satisfactory when I-CVI values reached 0.78 or higher and the S-CVI was at least 0.80 [43].

#### Construct validity

The full dataset (*N* = 558) was randomly split into two equal subsamples to conduct exploratory factor analysis (EFA; *n* = 278) and confirmatory factor analysis (CFA; *n* = 278). Prior to EFA, sampling adequacy was verified using the Kaiser–Meyer–Olkin (KMO) measure, with values exceeding 0.60, along with a significant Bartlett’s test of sphericity (*p* < 0.05) [40]. Factors were extracted using principal component analysis with varimax rotation, retaining components with eigenvalues greater than 1 and discarding items with factor loadings below 0.50 [44]. Model fit in CFA was evaluated using multiple goodness-of-fit indices, including CMIN/DF, RMSEA, CFI, IFI, NFI, and TLI [45]. Prior to model evaluation, a priori screening criteria were specified. Standardized factor loadings ≥ 0.50 were considered acceptable for item retention [44]. Model modifications were guided by modification indices (MI > 10) and were applied only when theoretically meaningful. Correlated error terms were permitted solely between items within the same latent construct and only when supported by conceptual overlap. No cross-loadings or post hoc structural paths were added [46]. These standards were applied uniformly to preserve both the conceptual coherence and the parsimony of the measurement model.

### Reliability analysis

The reliability of the Chinese S-AKO was examined by assessing internal consistency, split-half reliability, and test–retest stability [47]. Cronbach’s α coefficients were computed for both the overall scale and its subscales, with values exceeding 0.70 considered indicative of satisfactory reliability [48]. Split-half reliability was determined by calculating scale scores obtained from two complementary item sets. Test–retest stability was examined over a one-week interval in a separate group of participants (*n* = 30), with intraclass correlation coefficients (ICC) above 0.61 regarded as indicative of acceptable temporal reliability [47].

## Results

### Cross-cultural adaptation results of the S-AKO

In collaboration with a panel of 14 experts, the research team refined the wording of several items to improve the questionnaire’s cultural and contextual relevance while preserving the original item count. Pilot testing confirmed that the items were clearly expressed and appropriately captured the intended constructs, and thus no substantive revisions were deemed necessary. The expert-informed refinements included the following modifications: Item 3 was revised from “The care recipient refuses to accept assistance with oral care” to “I think the care recipient is very likely to refuse my assistance with oral care.” Item 4 was changed from “I can spare the necessary time to perform oral care” to “I can provide the time needed for oral care.” Item 5 was modified from “I have sufficient knowledge to carry out standardized oral care” to “I have enough knowledge to carry out standardized oral care.” Item 7 was adjusted from “I can give appropriate oral care advice to care recipients who need to perform oral care by themselves” to “To caregivers who want to take care of their oral care themselves, I can give appropriate oral care advice.” All revisions were reviewed and approved by the original developers of the instrument to ensure that the conceptual intent and theoretical meaning of each item remained intact.

### Descriptive statistics

Of the 574 questionnaires distributed and subsequently collected, meeting the predetermined sample size requirement. Following data screening, 16 responses were excluded, and 558 valid questionnaires were retained for the final analysis, corresponding to a valid response rate of 97.21%. The demographic profiles of undergraduate intern nursing students and RNs are summarized in Tables 1 and 2. Descriptive comparisons between the two groups indicated significant differences in most characteristics (Table 3): Gender (χ² = 11.990, *p* < 0.001), Age (t = -29.535, *p* < 0.001), Residence (χ² = 350.970, *p* < 0.001), Passion for nursing (χ² = 28.167, *p* < 0.001), Oral health care assessment tools (χ² = 11.104, *p* < 0.05), and Oral healthcare training (χ² = 15.068, *p* < 0.001). Self-rated oral health was comparable between groups, with no significant difference detected (χ² = 3.361, *p* > 0.05). These comparisons demonstrate that while nursing interns and RNs differ in demographic and professional characteristics, the groups were pooled for factor analyses to examine the overall instrument structure.

**Table 1:** Demographic data of undergraduate intern nursing students (*n* = 318)

Variables	Category	EFA (*n* = 162, %)	CFA (*n* = 156, %)
Sex	Male	26 ( 16.05)	26 (16.67)
	Female	136 (83.95)	130 (83.33)
Age, years	21.42 ± 1.72		
Department	Emergency	18 (11.11)	20 (12.82)
	ICU	17 (10.49)	27 (17.31)
	Internal medicine	25 (15.43)	23 (14.74)
	Surgery	29 (17.90)	34 (21.79)
	Pediatrics	22 (13.58)	8 (5.13)
	Obstetrics and Gynecology	17 (10.49)	6 (3.85)
	Operating room	20 (12.35)	16 (10.26)
	Psychiatry	5 (3.09)	10 (6.41)
	Others	9 (5.56)	12 (7.69)
Place of residence	Rural	55 (33.95)	48 (30.77)
	Urban-rural fringe	13 (8.02)	16 (10.26)
	Urban	94 (58.02)	92 (58.97)
Passion for nursing	Strong	98 (60.49)	84 (53.85)
	Medium	50 (30.86)	54 (34.62)
	Low	14 (8.64)	18 (11.54)
Oral health care assessment tools	Have used	13 (8.02)	16 (10.26)
	Heard, but haven’t used	80 (49.38)	68 (43.59)
	Haven’t heard and used	69 (42.59)	72 (46.15)
Oral health care training	Yes	35 (21.60)	33 (21.15)
	No	127 (78.40)	123 (78.85)
Self-rated oral health	Good	61 (37.65)	70 (78.85)
	Fair	83 (51.23)	79 (50.64)
	Bad	18 (11.11)	7 (4.49)

**Table 2:** Demographic data of RNs (*n* = 240)

Variables	Category	EFA (*n* = 117, %)	CFA (*n* = 123, %)
Sex	Male	6 (51.28)	10 (8.13)
	Female	111 (94.87)	113 (91.87)
Age, years	22 ~ 30	26 (22.22)	32 (26.02)
	31 ~ 40	67 (57.27)	58 (47.15)
	>40	24 (20.51)	33 (26.83)
Educational level	Associate Degree	4 (3.42)	6 (4.88)
	undergraduate degree	105 (89.74)	113 (91.87)
	Postgraduate degree	8 (6.84)	4 (3.25)
Place of residence	Rural	4 (3.42)	9 (7.32)
	Urban-rural fringe	5 (4.27)	7 (5.69)
	Urban	108 (92.31)	107 (86.99)
Working experience, years	<1	7 (5.98)	6 (4.88)
	1 ~ 3	4 (3.42)	7 (5.69)
	4 ~ 5	14 (11.97)	8 (6.50)
	6 ~ 15	54 (46.15)	63 (51.22)
	>15	38 (32.48)	39 (31.71)
Registered nurses	Registered nurse	9 (7.69)	11 (8.94)
	Senior nurse	31 (26.50)	36 (29.27)
	Nurse-in charge	57 (48.72)	55 (44.72)
	Associate chief nurse or above	20 (17.09)	21 (17.07)
Department	Emergency	6 (5.13)	9 (7.32)
	ICU	12 (10.26)	12 (9.76)
	Internal medicine	17 (14.53)	26 (21.14)
	Surgery	53 (45.30)	45 (36.59)
	Pediatrics	0 (0 )	2 (1.63)
	Obstetrics and Gynecology	8 (6.84)	5 (4.07)
	Operating room	2 (1.71)	0 (0)
	Others	19 (16.24)	24 (19.51)
Passion for nursing	Strong	91 (77.78)	96 (78.05)
	Medium	22 (18.80)	24 (19.51)
	Low	4 (3.42)	3 (2.44)
Oral health care assessment tools	Have used	14 (11.97)	30 (24.39)
	Heard, but haven’t used	57 (48.72)	52 (42.28)
	Haven’t heard and used	46 (39.32)	41 (33.33)
Oral health care training	Yes	39 (33.33)	48 (39.02)
	No	78 (66.67)	75 (60.98)
Self-rated oral health	Good	38 (32.48)	58 (47.15)
	Fair	63 (53.85)	51 (41.46)
	Bad	16 (13.68)	14 (11.38)

**Table 3:** Descriptive comparison of demographic and oral-health–related characteristics between undergraduate intern nursing students and RNs (*n* = 558)

Variables	Category	Nursing Interns (*n* = 318)	Registered Nurses (*n* = 240)	t/χ²	*p*-value
Gender, n	Male	52	16	11.990	*p* < 0.001
	Female	266	224		
Age, mean ± SD		21.42 ± 1.72	36.48 ± 7.75	-29.535	*p* < 0.001
Residence, n	Rural	103	13	350.970	*p* < 0.001
	Urban-rural fringe	29	12		
	Urban	186	215		
Passion for nursing, n	Strong	182	187	28.167	*p* < 0.001
	Medium	104	46		
	Low	32	7		
Oral health care assessment tools, n	Have used	29	44	11.104	*p* < 0.05
	Heard, but haven’t used	148	109		
	Haven’t heard and used	141	87		
Oral healthcare training, n	Yes	68	87	15.068	*p* < 0.001
	No	250	153		
Self-rated oral health, n (%)	Good	131	96	3.361	*p* > 0.05
	Fair	162	114		
	Bad	25	30		

### Item analysis

In the Chinese S-AKO, CR values (3.32–22.51) indicated adequate item discrimination, with all items demonstrating significant differences between extreme-score groups (*p* < 0.001). Item–total correlation coefficients varied between 0.311 and 0.731, supporting the retention of all items for further analysis

### Validity analysis

#### Content validity

Content validity was assessed by a panel of 14 experts. The I-CVI and S-CVI values were 0.857 and 0.938, respectively, indicating satisfactory content validity of the scale.

### Exploratory factor analysis

Bartlett’s test of sphericity was statistically significant (χ² = 622.330, *p* < 0.001), and the KMO value reached 0.883, confirming the adequacy of the dataset for factor extraction. Principal component analysis with varimax rotation yielded three factors with eigenvalues above 1, collectively explaining 80.941% of the variance. All retained items loaded strongly on their respective factors, with loadings exceeding 0.50. In line with the original scale structure, the three factors were labeled as “Attitudes to Oral Hygiene,” “Implementation Possibilities,” and “Knowledge of Importance.” All thirteen items demonstrated factor loadings ≥ 0.50 with no evidence of cross-loadings; consequently, all items were retained for subsequent analyses, and the detailed factor analytic results are summarized in Table 4.

**Table 4:** Rotated factor structure of the questionnaire (*n* = 279)

Item	F1	F2	F3
Q12. Oral physiological functions (e.g., chewing, swallowing, speaking).	0.908	0.150	-0.123
Q11. Characteristics of a healthy mouth.	0.899	0.163	-0.079
Q13. Oral socio-psychological functions (e.g., appearance, well-being).	0.887	0.171	-0.116
Q9. Diseases affecting the oral health.	0.887	0.129	-0.082
Q8. Oral care assistive products.	0.880	0.234	-0.115
Q10. Various artificial (prosthetic) dental substitutes.	0.777	0.285	-0.002
Q5. I have enough knowledge to carry out standardized oral care.	0.142	0.922	0.018
Q6. I have the appropriate auxiliary tools needed to perform standardized oral care.	0.138	0.907	0.076
Q7. To caregivers who want to take care of their oral care themselves, I can give appropriate oral care advice.	0.255	0.890	-0.028
Q4. I can provide the time needed for oral care.	0.318	0.781	-0.097
Q2. I think it is practically difficult to carry out oral care.	-0.082	-0.053	0.905
Q3. I think the care recipient is very likely to refuse my assistance with oral care.	-0.077	0.040	0.872
Q1. I think it feels nasty to provide oral care for others.	-0.132	0.003	0.861
Cumulative variance contribution rate	37.084%	62.550%	80.941%

F1 Knowledge of importance; F2 Implementation possibilities; F3 Attitudes to oral hygiene

### Confirmatory factor analysis

**Fig. 1:**
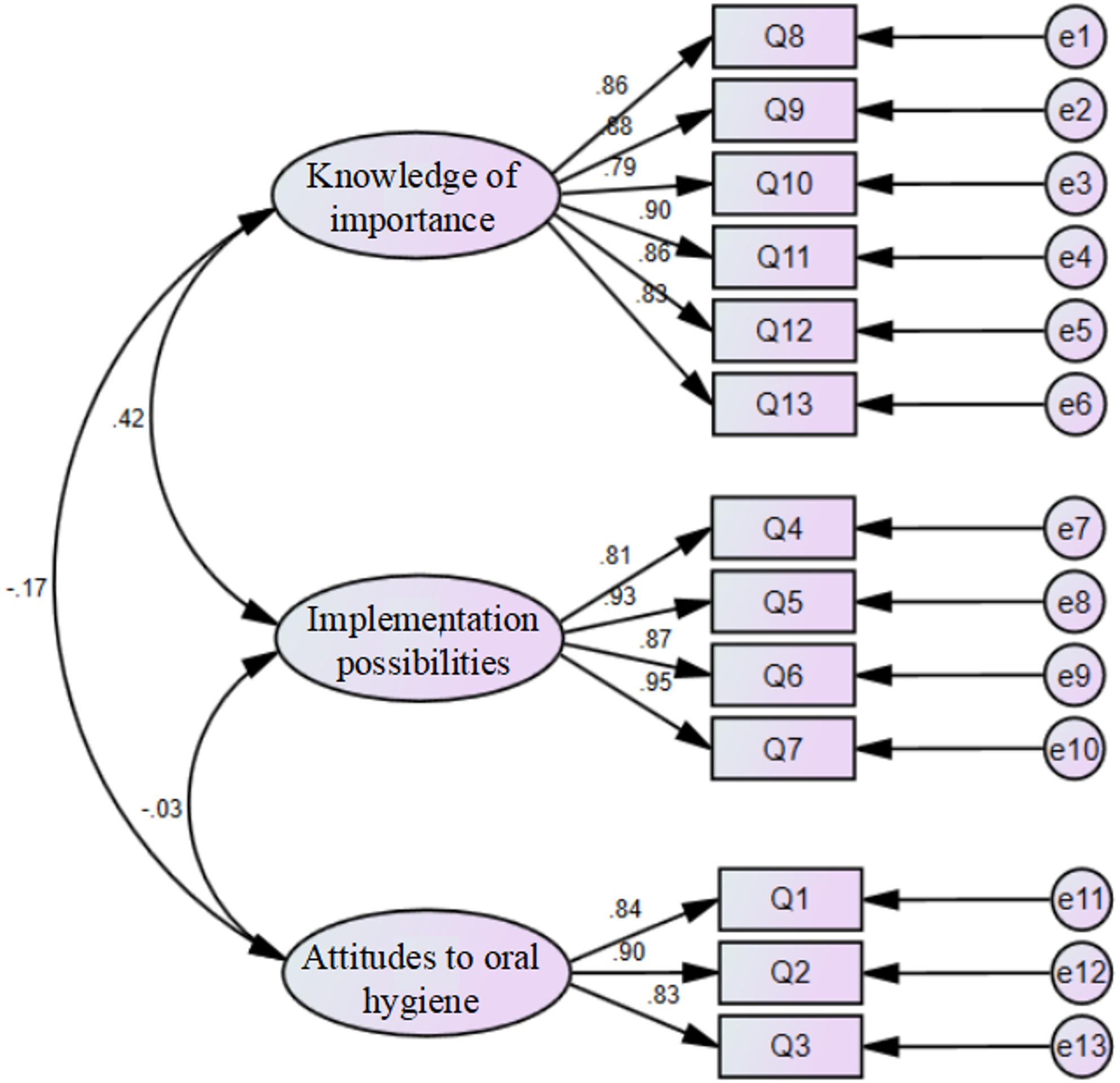
Three-factor CFA model of the S-AKO (*n* = 279)

The CFA results (Fig. 1) indicated that the final model fit the data well, with all key indices (CMIN/DF = 2.466, RMSEA = 0.073, CFI = 0.970, IFI = 0.970, NFI = 0.951, TLI = 0.962) meeting or surpassing recommended thresholds, confirming the suitability of the AMOS 24.0 measurement model.

### Reliability analysis

Internal consistency of the Chinese S-AKO was robust, with a total scale Cronbach’s α of 0.810 and subscale α coefficients from 0.879 to 0.946, indicating the measure is reliably consistent. Stability over time was supported by a test–retest coefficient of 0.923, and split-half reliability of 0.726 further indicated consistent measurement.

## Discussion

In this study, the short-form Swedish S-AKO questionnaire was translated into Chinese following established cross-cultural adaptation procedures and refined through expert consultation [49]. Psychometric analyses demonstrated acceptable item discrimination, reliability, test–retest stability, and validity. These findings directly support the study aim of establishing a brief, psychometrically sound instrument for assessing nursing professionals’ oral health–related knowledge and attitudes within the Chinese educational and clinical context. CFA identified and verified a three-factor structure, providing empirical support for the underlying conceptual model of oral healthcare competence applied in this study.

Validity reflects the extent to which a tool effectively captures the target construct, usually examined through content and construct validity [50]. Based on expert ratings for clarity, relevance, and representativeness, content validity was demonstrated, with all I-CVI and S-CVI values above the recommended cutoff. This result indicates strong expert consensus that the Chinese S-AKO adequately captures key theoretically grounded components of nursing oral healthcare competence, namely knowledge, attitudes, and perceived feasibility of implementation, which have been consistently emphasized in prior oral health and nursing education literature [51]. In addition, based on expert feedback and pilot testing, several linguistic and contextual adjustments were made to ensure semantic equivalence and alignment with Chinese nursing education and clinical practice, enhancing the instrument’s contextual appropriateness and applicability across settings.

The three-factor model was consistently supported by EFA and CFA, confirming the conceptualization of oral healthcare competence as a multidimensional construct encompassing knowledge, attitudes, and perceived implementation possibilities. The empirical confirmation of this structure indicates that these components are distinct yet interrelated, providing a theory-informed basis for interpreting assessment results and guiding targeted educational interventions.

The Chinese S-AKO demonstrated satisfactory internal consistency and stability over time, with Cronbach’s α, split-half reliability, and test–retest reliability all exceeding recommended thresholds, supporting its use in both cross-sectional and longitudinal evaluations of nursing competencies in oral healthcare.

In recent years, oral healthcare has received increasing attention in China, yet empirical research on nursing professionals’ oral health–related knowledge and attitudes remains limited [52]; however, few studies have focused on oral healthcare education among nursing professionals. Previous research [53, 54] has identified insufficient oral health knowledge, attitudes, and self-efficacy as major barriers to providing high-quality oral care, emphasizing the need for enhanced oral health education. The present study addresses this gap by validating a concise, theory-informed instrument that provides an empirical means to operationalize and assess these barriers, moving beyond purely conceptual or descriptive accounts of oral healthcare competence among nursing professionals. In China, nursing professionals, especially RNs, receive general nursing education with limited oral health conten [55], and oral care often receives lower priority due to heavy workloads and competing clinical demands [56]. Within this context, the Chinese S-AKO enables systematic identification of specific competency gaps relevant to education and clinical training. Together, the three dimensions reflect a theory-based understanding of oral healthcare competence as shaped by knowledge, attitudinal readiness, and perceived feasibility within clinical practice, highlighting that deficits extend beyond knowledge alone.

Overall, after thorough cross-cultural adaptation and psychometric testing, the Chinese S-AKO exhibited robust cultural applicability, reliability, and validity. With only 13 items and requiring around three minutes to complete, the instrument imposes a low response burden, making it highly suitable for use in educational and clinical settings. Its brevity and psychometric robustness enhance feasibility for routine assessment in busy clinical environments, thereby supporting evidence-informed oral healthcare education and practice.

### Limitations

First, the single-timepoint study design precludes claims about the tool’s long-term stability and predictive validity; longitudinal research is needed to address this. Second, depending on self-reported responses may introduce social desirability and recall biases; subsequent research could incorporate mixed-methods or observational approaches to mitigate these limitations. Third, this study was confined to hospital settings. Given the increasing shift of healthcare services toward community and home-based care, future research should expand to include these settings. Fourth, although nursing interns and registered nurses differ in clinical exposure and oral-care responsibilities, factor analyses were conducted on the pooled sample to establish the instrument’s overall factorial validity. While this enhanced statistical power, it did not allow formal testing of measurement invariance across professional groups, which should be examined in future research. Finally, the criterion validity could not be assessed because a recognized gold-standard instrument is not available in China,, underscoring the need for such a benchmark in future studies.

## Conclusions

The Chinese S-AKO questionnaire exhibits robust validity and reliability, serving as a robust instrument for assessing nursing professionals’ knowledge and attitudes toward oral health. It holds substantial value for both research and clinical education, as it enables educators to identify learners’ strengths and deficiencies in oral health competencies and to evaluate the effectiveness of related training initiatives. Incorporating this questionnaire into nursing curricula and continuing education programs can help reveal specific educational gaps, guide the refinement of teaching content, and promote the development of targeted and evidence-based training interventions. Moreover, its use in clinical settings can help educators and nursing managers monitor professional growth, reinforce the integration of oral health into daily nursing care, and foster reflective learning. Ultimately, its application may advance oral health education, enhance RNs’ capacity to provide patient-centered oral care, and contribute to improved holistic health outcomes.

## Supplementary Information

Supplementary Material 1.

Supplementary Material 2.

## Data Availability

The data supporting the findings of this study can be obtained from the corresponding author upon reasonable request.

## Abbreviations

S-AKO: A short-form version of the Swedish “Attitudes to and Knowledge of Oral Health” questionnaire
RNs: Registered nurses
SPSS: Statistical Product and Service Solutions
AMOS: Analysis of moment structure
KMO: Kaiser-Meyer-Olkin
EFA: Exploratory factor analysis
CFA: Confirmatory factor analysis
I-CVI: Item-level content validity index
S-CVI: Scale-level content validity index
ICC: Intraclass correlation coefficient
RMSEA: Root mean square error of approximation
CFI: Comparative fit index
TLI: Tucker Lewis index
NFI: Normed fit index
IFI: Incremental fit Index
