# Supplementary material for: Psychometric validation of the short-form Swedish “attitudes to and knowledge of oral health” (S-AKO) questionnaire in Chinese nursing professionals: a cross-sectional study
Source: BMC Oral Health. 2026 Mar 13;26:710. doi: 10.1186/s12903-026-08076-1 (PMC13101299; doi:10.1186/s12903-026-08076-1)
Supplement: Supplementary file 1 — Supplementary Material 1. [file 12903_2026_8076_MOESM1_ESM.doc]

**Appendix 1 S-AKO questionnaire item-level overview**

| **Dimensions** | **Item focus (summary)** | **Core competency addressed** |
| --- | --- | --- |
| Attitudes to Oral Hygiene | Value placed on daily oral care for dependent older adults | Professional responsibility; preventive orientation |
| Implementation Possibilities | Perceived feasibility under workload and resource constraints | Clinical self-efficacy; system-level feasibility |
| Knowledge of Importance | Understanding links between oral health and systemic health | Foundational oral-health literacy |
