# Supplementary material for: Psychometric validation of the short-form Swedish “attitudes to and knowledge of oral health” (S-AKO) questionnaire in Chinese nursing professionals: a cross-sectional study
Source: BMC Oral Health. 2026 Mar 13;26:710. doi: 10.1186/s12903-026-08076-1 (PMC13101299; doi:10.1186/s12903-026-08076-1)
Supplement: Supplementary file 2 — Supplementary Material 2. [file 12903_2026_8076_MOESM2_ESM.doc]

**Appendix 2** AKO questionnaire

**Attitudes to oral hygiene**

Item 1- I think it feels nasty to take care of other people's mouths

Item 2- I think oral care is part of my job duties

Item 3- I think it is practically difficult to perform oral care

Item 4- The caregiver refuses to receive help with oral care

**Implementation possibilities**

**What opportunities do you think you have when it comes to offering oral care to the healthcare provider you are responsible for?**

Item 5- I can take the time needed to provide oral care

Item 6- I have enough knowledge to perform proper oral care

Item 7- I have appropriate aids for the implementation of proper oral care

Item 8- I know how to practically perform oral care

Item 9- To caregivers who want to take care of their oral care themselves, I can give appropriate oral care advice

Item 10- By actively informing "reluctant" caregivers, I can in the long run get them to accept help with oral care

**Knowledge of importance**

**What skills do you think are important for being able to perform good oral care?**

Item 11- Assistive products and oral care

Item 12- Diseases affecting the oral cavity

Item 13- Various artificial (prosthetic) dental substitutes

Item 14- What the healthy oral cavity looks like

Item 15- Oral physiological function (e.g., chewing, swallowing, speech)

Item 16- The psychosocial function of the oral cavity (e.g., appearance, well-being)
